# The potential of virtual triage AI to improve early detection, care acuity alignment, and emergent care referral of life-threatening conditions

**DOI:** 10.3389/fpubh.2024.1362246

**Published:** 2024-05-13

**Authors:** George A. Gellert, Aleksandra Kabat-Karabon, Gabriel L. Gellert, Joanna Rasławska-Socha, Stanislaw Gorski, Tim Price, Kacper Kuszczyński, Natalia Marcjasz, Mateusz Palczewski, Jakub Jaszczak, Irving K. Loh, Piotr M. Orzechowski

**Affiliations:** ^1^Infermedica, Inc, San Antonio, TX, United States; ^2^Infermedica, Inc, Wroclaw, Poland; ^3^Infermedica, Inc, Toronto, Canada; ^4^Department of Medical Education, Jagiellonian University Medical College, Kraków, Poland; ^5^Infermedica Inc, London, United Kingdom; ^6^Pediatric Surgery and Urology Department, Wroclaw Medical University, Wroclaw, Poland; ^7^Ventura Heart Institute, California, Thousand Oaks, CA, United States

**Keywords:** virtual/digital clinical triage/care referral, artificial intelligence, care delay, early disease detection, asthma, pneumonia, myocardial infarction, stroke

## Abstract

**Objective:**

To evaluate the extent to which patient-users reporting symptoms of five severe/acute conditions requiring emergency care to an AI-based virtual triage (VT) engine had no intention to get such care, and whose acuity perception was misaligned or decoupled from actual risk of life-threatening symptoms.

**Methods:**

A dataset of 3,022,882 VT interviews conducted over 16 months was evaluated to quantify and describe patient-users reporting symptoms of five potentially life-threatening conditions whose pre-triage healthcare intention was other than seeking urgent care, including myocardial infarction, stroke, asthma exacerbation, pneumonia, and pulmonary embolism.

**Results:**

Healthcare intent data was obtained for 12,101 VT patient-user interviews. Across all five conditions a weighted mean of 38.5% of individuals whose VT indicated a condition requiring emergency care had no pre-triage intent to consult a physician. Furthermore, 61.5% intending to possibly consult a physician had no intent to seek emergency medical care. After adjustment for 13% VT safety over-triage/referral to ED, a weighted mean of 33.5% of patient-users had no intent to seek professional care, and 53.5% had no intent to seek emergency care.

**Conclusion:**

AI-based VT may offer a vehicle for early detection and care acuity alignment of severe evolving pathology by engaging patients who believe their symptoms are not serious, and for accelerating care referral and delivery for life-threatening conditions where patient misunderstanding of risk, or indecision, causes care delay. A next step will be clinical confirmation that when decoupling of patient care intent from emergent care need occurs, VT can influence patient behavior to accelerate care engagement and/or emergency care dispatch and treatment to improve clinical outcomes.

## Introduction

Life-threatening illness requiring emergency care accounts for 14.0% of emergency department (ED) visits in the US, and benefits from the earliest possible detection and care referral to improve patient outcomes and reduce hospital length of stay and care costs (1, 2). If patient understanding/self-perception underestimates actual acuity and risk indicated by symptoms and past medical history, patients may delay care seeking (3–5). Delayed care results in avoidable mortality and drives higher acuity care utilization that is more costly than might have been needed had the patient sought care earlier (3, 6).

Symptomate is a current generation digital virtual triage (VT) engine or “symptom checker” that leverages artificial intelligence (AI) to provide access to authoritative information services beyond physician office hours on a 24/7/365 basis from any device with internet connectivity. VT can avert long ED waits or travel, and helps when individuals are uncertain if symptoms warrant medical attention or what kind of care to seek, or if symptoms worsen. The VT interview asks questions about age, sex, symptoms, medical history, risk factors, and medications and, processing responses on a current basis, selects the next most relevant question via Bayesian probabilities, simulating the manner in which a human clinician processes information. After evaluating all patient data, the VT inference engine computes the probabilities of likely conditions using a statistical algorithm for advanced symptom assessment informed by an underlying medical knowledge base. The VT AI advises the patient-user of probable causes, illness severity, and recommended acuity level of medical care. VT is superior to internet browser search because patients are evaluated systematically to identify/convey a care acuity level using clinical AI algorithms validated by physicians. VT AI improves upon traditional clinical triage protocols or decision trees in its ability to dynamically calculate the probability of possible conditions and adjust the patient interview accordingly. However, VT results are not diagnostic and do not constitute medical consultation, providing information and guidance only. Users are advised to seek immediate medical attention when the VT engine determines that a threat to life exists.

Critical to maximizing the clinical and public health impact of VT AI is identifying functional areas where it can enhance existing diagnostic processes and capabilities. It is unknown whether AI-based VT can augment existing healthcare system capabilities for early identification, care acuity alignment, and rapid care referral of individuals whose self-perception of disease acuity/risk is inaccurate when serious morbidity is actually evolving. Patient delay in seeking urgent care across life-threatening diseases increases mortality (3, 7). When patient perception-understanding of symptoms/risk is decoupled from high actual risk of a severe clinical outcome, if identified reliably by VT in real-time, ED care engagement can be accelerated. VT may thus offer a new opportunity for early life-threatening disease detection, enabling improved, more rapid care acuity alignment and ED referral. VT could enhance existing healthcare system capabilities and efforts to reduce preventable disease sequelae and death. This study evaluates decoupling of actual care acuity need from patient care intent/risk self-perception as detected by a clinically validated VT engine available cost-free online. We examine data on patient perception-actual risk decoupling during severe symptom onset of five leading causes of U.S. hospital admission and death.

## Methods

### Objective

Evaluate the extent to which patient-users reporting symptoms of severe, acute conditions classified by an AI-based VT engine as warranting emergency care had no pre-VT triage intention to engage appropriate ED care. The extent to which decoupling of patient care intent from actual clinical need occurs for five prevalent diseases could imply a potential real-time use of VT to improve early detection and care acuity alignment and accelerate more effective care referral for emergent conditions.

### VT technology platform and data source

The Infermedica Symptomate VT engine is designed for general public use and completes evidence-driven analyses informed by over 800 diseases, 1,500 symptoms, and 200 risk factors. Leveraging AI, machine learning, and natural language processing, VT evaluates symptoms reported by patient-users, suggesting the most probable conditions matching the presentation and history, and refers to the most clinically appropriate and safest possible care. There are no prescribed interview pathways, and in light of new information reported, the VT AI explores various clinical queries and hypotheses (as physicians do). Before VT, patient-users are asked about their care intention. The VT interview concludes with an analysis of the reported symptoms and a recommendation to pursue one of three levels of care acuity: self-care, consult a primary care or specialist physician on an outpatient basis, proceed to an ED or call an ambulance for ED transport.

Virtual triage technologies (including Symptomate) are considered medical device class I in Europe, according to Medical Device Directive (93/42/EEC) and in the US, under the Food, Drug & Cosmetic Act. The FDA currently exercises enforcement discretion, which means that virtual triage technology is not required to comply with FDA regulations related to medical devices.

Data were extracted from VT episodes engaged by patient-users of the Symptomate VT application, a stand-alone VT engine not integrated with/implemented within a health system, and available through the Infermedica website or as a mobile application. Symptomate is available in 24 languages. Over 17 million Symptomate evaluations have been completed since 2012.

### VT engine clinical validity

AI-based VT engines require rigorous validation to ensure safety and minimize mistriage, and focus on common diseases by design, with AI built to overtriage to emergency care rather than possibly miss a patient with acute care need. VT accuracy varies across clinical specialties and settings, as determined by the depth of disease-specific data used to train the triage AI. VT validity has been evaluated using clinical vignettes prepared by physicians of various patient symptomatic presentations in different clinical settings (8–11). Symptomate provides safe recommendations in 97.8% of instances (9). VT engines may perform poorly detecting low prevalence conditions or presentations in atypical clinical settings (8). Published studies, while providing a point in time comparison, become quickly outdated due to the rapid evolution of AI-based VT. For example, since the analysis conducted by 2019 Gilbert et al., (9) there have been 29 releases of the Symptomate medical content model and 17 updates to core functionality, including new epidemiological models and triage algorithms, each improving accuracy and safety. In a recent study of 8,008 patient-users of the Infermedica virtual triage engine in an ambulatory care network, 2,830 or 35.0% changed their healthcare seeking action from their initial pre-VT care intent as a result of the VT evaluation and recommendation, indicating that VT output can alter the care seeking behavior of patient-users (12).

### Sample selection and eligibility criteria

**Figure 1:**
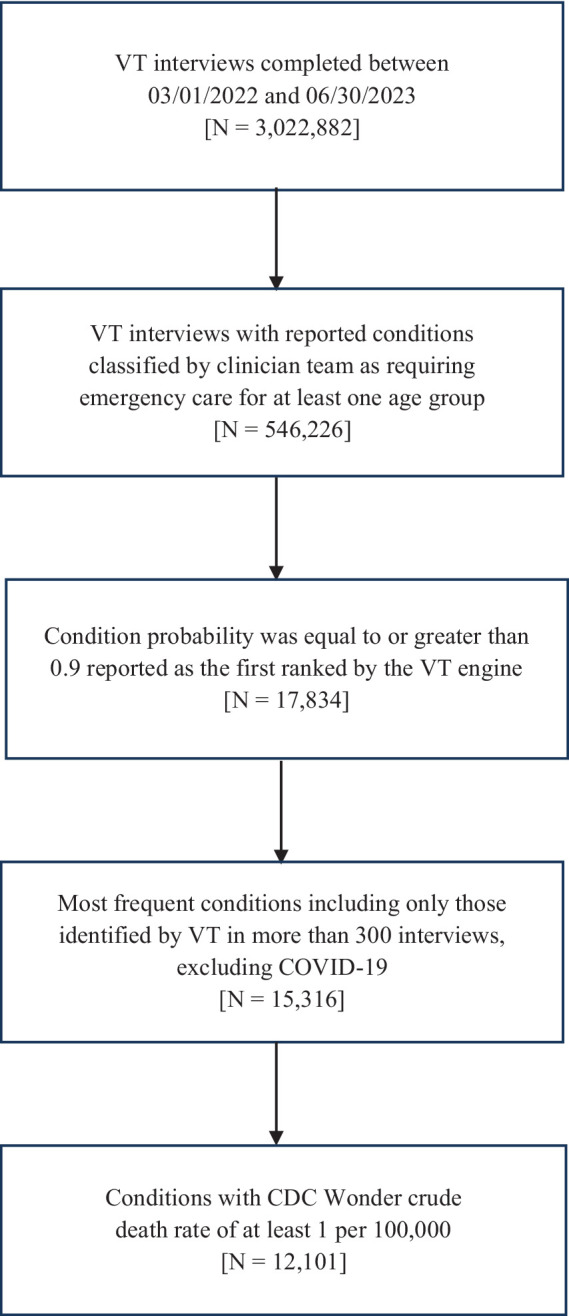
Flow chart of virtual triage patient-user eligibility for study inclusion.

Symptomate VT interviews were selected using the following criteria: (1) interview completed during a 16-month period of March 2022–June 2023; (2) first ranked condition classified as requiring emergency care; (3) condition probability equal or greater than 0.90 as the first ranked by VT; (4) conditions with minimum 300 interviews; and (5) conditions with crude death rate of 1 per 100,000 and above in CDC Wonder (13). COVID-19 was excluded. Only five conditions with high potential severity/mortality met these eligibility criteria, including myocardial infarction (MI), stroke, asthma, pneumonia, and pulmonary embolism (PE). While the VT engine uses the term/variable “myocardial infraction” in its logic, language used in the VT patient interface was adapted to ensure public comprehension (e.g., “heart attack”). Ischemic stroke (including cerebellar strokes) and hemorrhagic stroke (both subarachnoid and intracerebral) were collapsed into a single category for analysis. Figure 1 conveys the sequential application of eligibility criteria to patient-user interview selection.

### Data captured and analyses completed

All data was collected through the Symptomate VT engine database. VT interviews were completed anonymously with no individual identifiers recorded beyond age, gender, and language. Patient-users conveyed informed consent for using their anonymized data in the aggregate devoid of personal identifiers. A dataset of 3,022,882 patient-users was evaluated to investigate the extent of decoupling of patient-user pre-triage care intent and VT-identified high acuity conditions warranting emergency care. The pre-triage care intention survey provided response options of doing nothing; self-care/recover at home; consult a doctor; emergency care with or without ambulance; and not sure/do not know/none. While care intent after VT guidance was queried, completion rates were insufficient to support meaningful analysis.

Pre-interview intent responses of doing nothing, self-care/recovering at home, were grouped and classified as non-professional care intent. Intent to consult an outpatient physician, do nothing, and self-care/recovering at home were grouped and classified as non-urgent care intent. For each condition, frequency of responses indicating pre-triage intent of non-professional care and non-emergent care were determined and assessed for systematic variation by demographics. Google Sheets was used to complete chi-squared tests, assessing for statistically significant differences between demographic groups (age, gender, and language) by care intent responses at the *p* = 0.05 level. To calculate the aggregated level of indicators in Table 1 encompassing various patient care intents across all conditions included in the analysis, weighted arithmetic means were calculated using each condition’s respective prevalence as its weighted value. Weighted means were calculated across the five diseases by multiplying the number of VT interviews for each condition by the percentage of interviews with stated intent for that condition, then summing and dividing by the sum of interviews.

**Table 1:** Decoupling of patient care intent from virtual triage care referral for five causes of us morbidity and mortality.

Clinical condition	Total number of interviews with patient intent completed	Patients indicated non-professional care intent (do nothing, recover at home/self-care)	Patients indicated non-emergent care intent (consult outpatient physician, do nothing, recover at home/self-care)	Patients indicated intent to seek emergency care before virtual triage
Asthma Exacerbation	3,730	39.6%	63.4%	6.1%
Pneumonia	4,873	42.5%	65.8%	5.8%
Pulmonary Embolism	526	36.5%	53.0%	18.0%
Myocardial Infarction	2,189	32.3%	54.4%	10.0%
Stroke^1^	783	27.6%	52.4%	15.9%
All Conditions^2^	12,101	38.5%	61.5%	7.8%

^1^Stroke tabulations include ischemic, hemorrhagic, and cerebellar strokes plus subarachnoid hemorrhages.

^2^Percentages in bottom row totals are weighted mean values.

Because data were derived from general public Symptomate use and not patient-users within a healthcare system, it was impossible to follow patients’ clinical course to confirm acuity and clinical outcomes.

### Adjustment for overtriage to emergency care

VT technology errs on the side of over-caution in care referral because of the potentially serious harm/injury to patients if VT under-refers to ED/ambulance, favoring care referral sensitivity over specificity by design and ethical imperative. Analyses of Symptomate over-referral have indicated that approximately 13% of ED recommendations are over-referrals, which has been declining as its VT AI advances (14). Symptomate performance was compared with widely used teletriage clinical decisioning protocols using clinical vignettes. VT over-referred 12% of patients to ED versus 26% for a leading telephone triage product (Schmitt-Thompson) (15). To enhance precision of estimates of decoupling of patient pre-triage care intent from VT acuity/referral output, intention rates were adjusted for overtriage to ED by a factor of 13%.

## Results

### Decoupling of patient pre-triage care intent and VT acuity assessment

Intent data were obtained for 12,101 VT patient-user interviews. Across all five conditions, a weighted mean of 38.5% of individuals whose VT indicated a condition requiring emergency care had no pre-triage intent to consult a physician. Furthermore, 61.5% intending to possibly consult a physician had no intent to seek emergency medical care (Table 1). The low frequency of patient-users intending before VT to seek emergency care is striking and may reflect the use of VT quite early in symptom evolution, presumably increasing with an experience of more severe symptoms. In addition, individuals with severe symptoms may bypass VT use in favor of telephonic consultation or in-person visit to an ED.

Table 2 presents adjusted rates factoring in VT safety over-triage to ED and shows that after allowing for a 13% over referral to ED, a weighted average of 33.5% of patient-users across conditions had no intent to seek professional care. One half (53.5%) had no intent to seek emergency care.

**Table 2:** Patient care intent-virtual triage care referral decoupling adjusted for AI safety overtriage to emergency department.

Clinical condition	Patients indicated non-professional care intent (do nothing, recover at home/self-care)	Patients indicated non-professional care intent adjusted for virtual triage safety overtriage to emergency department^1^	Patients indicated non-emergent care intent (consult outpatient physician, do nothing, recover at home/self-care)	Patients indicated non-emergent care intent adjusted for virtual triage safety overtriage to emergency department^1^
Asthma Exacerbation	39.6%	34.5%	63.4%	55.2%
Pneumonia	42.5%	37.0%	65.8%	57.2%
Pulmonary Embolism	36.5%	31.8%	53.0%	46.1%
Myocardial Infarction	32.3%	28.1%	54.4%	47.3%
Stroke^2^	27.6%	24.0%	52.4%	45.6%
All Conditions^3^	38.5%	33.5%	61.5%	53.5%

^1^Adjusted for estimated 13% virtual triage engine overtriage with referral to emergency care in order to maximize patient safety (favoring medical emergency detection sensitivity over specificity).

^2^Stroke tabulations include ischemic, hemorrhagic, and cerebellar strokes plus subarachnoid hemorrhages.

^3^Percentages in bottom row totals are weighted mean values.

### Demographic analyses of pre-triage care intent by age, gender, and language

With respect to demographics, all age groups were statistically significantly different from one another (*p* = 0.05). The youngest (under 18 are completed by a parent or adult caretaker) and the eldest age groups were more likely to express intent to seek appropriate emergency care for symptoms, with those 18–44 years least likely to, as shown in Table 3.

**Table 3:** Patient-user pre-VT intent by age.

Age	Patients indicated non-professional care intent (do nothing, recover at home/self-care)	Patients indicated non-emergent care intent (consult outpatient physician, do nothing, recover at home/self-care)	Patients indicated intent to seek emergency care	Number of interviews (age category total percentages)
0–17	119 (32.0%)	193 (51.9%)	54 (19.1%)	372 (3.1%)
18–29	2,815 (40.0%)	4,375 (62.2%)	325 (6.1%)	7,037 (58.2%)
30–44	1,089 (37.8%)	1,809 (62.8%)	164 (7.4%)	2,879 (23.8%)
45–59	377 (37.1%)	631 (62.0%)	73 (9.1%)	1,017 (8.4%)
60–74	178 (33.9%)	291 (55.4%)	56 (14.2%)	525 (4.3%)
75+	83 (30.6%)	148 (54.6%)	50 (22.0%)	271 (2.2%)
Total	–	–	–	12,101 (100.0%)

Age stratum percentages are calculated relative to the particular age group, where the denominator is the number of patient-users expressing that intent or intentions within each age group.

Regarding respondent gender (Table 4), men were more likely (40.7%) than women (37.6%) to indicate a non-professional care intent (*p* = 0.05). Men were also more likely to state an intent to seek emergency care (9.3%) than women (7.1%) (*p* = 0.05). The sample breakdown of patient-user respondents by gender, with women comprising over two-thirds of VT interviews, reflects the pattern of all Symptomate users (16). Overall, the lack of perceived urgency of symptoms is similar between genders.

**Table 4:** Patient-user pre-VT intent by gender.

Gender	Patients indicated non-professional care intent do nothing, recover at home/self-care	Patients indicated non-emergent care intent (consult outpatient physician, do nothing, recover at home/self-care)	Patients indicated intent to seek emergency care	Number of interviews
Female	3,160 (37.6%)	5,140 (61.1%)	453 (7.1%)	8,413 (69.5%)
Male	1,501 (40.7%)	2,307 (62.6%)	269 (9.3%)	3,688 (30.5%)
Total	–	–	–	12,101 (100.0%)

All percentages are calculated relative to the gender group, where the denominator is the number of patient-users expressing that intent or intentions for each gender.

Patient-users in each major language grouping (English, Spanish, French, German, Polish, Russian and other) were statistically significantly different from each other in every intent category (*p* = 0.05) (Table 5). Those completing VT in Spanish were more likely to express appropriate emergency care intent than patient-users in other language groups (10.2%). Those completing in French were lowest in reporting intent to engage emergency care at 4.7%, but were most inclined to engage non-emergent care including consulting an outpatient physician.

**Table 5:** Patient-user pre-VT intent by interview language.

Language of virtual triage use	Patients Indicated Non-professional care intent (do nothing, recover at home/self-care)	Patients indicated non-emergent care intent (consult outpatient physician, do nothing, recover at home/self-care)	Patients indicated intent to seek emergency care	Number of interviews
English	1,861 (36.2%)	2,925 (56.8%)	295 (8.0%)	5,147 (42.5%)
Spanish	864 (38.7%)	1,404 (62.8%)	185 (10.2%)	2,234 (18.5%)
French	725 (45.6%)	1,112 (70.0%)	63 (4.7%)	1,589 (13.1%)
German	324 (43.7%)	477 (64.4%)	29 (5.2%)	741 (6.1%)
Polish	233 (39.6%)	386 (65.6%)	36 (7.6%)	588 (4.9%)
Russian	153 (33.6%)	285 (62.6%)	25 (7.2%)	455 (3.8%)
Other Languages	501 (37.2%)	858 (63.7%)	89 (8.2%)	1,347 (11.1%)
Total	–	–	–	12,101 (100.0%)

All percentages are calculated relative to the language group, where the denominator is the number of patient-users expressing that care intent or intentions by each language.

## Discussion

Delays in care seeking during medical emergencies negatively impact patient outcomes, and may result from patients failing to understand, dismissing or underestimating serious symptoms, and/or attempting to self-treat serious clinical problems at home (3). Table 6 summarizes causes and impact of care delay for the five conditions evaluated. Early detection and prompt, acuity appropriate treatment are crucial in improving outcomes of MI, which killed 108,610 Americans in 2018 (13). Almost 48% of VT patient-users with likely MI understood their symptoms required physician care but not immediate/urgent care, yet 28.1% planned to recover at home with no medical care (ED overtriage adjusted). This aligns with national mortality data. Reducing MI care delays focuses on patient education about symptoms warranting early care seeking (3, 18, 19), and can be integrated with VT.

**Table 6:** Causes and impact of care delay for five life-threatening conditions.

Disease	Causes of detection/care delay	Impact of detection/care delay	Annual US mortality and morbidity
Myocardial Infarction	Engaging outpatient not emergency care (17, 19) Misinterpretation/underestimation of MI symptoms (3, 17, 19) Wait-and-see attitude/assumption symptoms will subside spontaneously (3) Seeking nonprofessional help (3) Attempting to self-medicate (3)	Every 30-min delay increases 1-year mortality 7.5% (18) 4-fold increased risk of in-hospital mortality (3)	Annual incidence 805,000 (20) 108,610 MI deaths in 2018 (13) 2018 crude death rate of 33.2 per 100,000; age-adjusted death rate of 27.0 per 100,000 (13)
Stroke	Engaging outpatient rather than emergency care (22) Unfamiliarity with stroke symptoms (23) Living alone (22, 23) Delayed decision to seek professional care (21, 23)	Increased mortality risk when no early suspicion of stroke (22) Every 60-min delay from symptom onset to treatment increases risk of reduced functional independence 5.3% and mortality 2.2% (24)	Annually >795,000 stroke cases (24) 147,810 stroke deaths in 2018 (13) Death rate of 45.2 per 100,000; age-adjusted rate of 37.1 per 100,000 (13)
Asthma	86% of severe attacks delay medical care and/or underestimate severity (7) Inability to obtain medical assistance outside working hours (5) Fatigue or feeling despondent/depressed (5)	45% of deaths for severe/final asthma attack due to lack of seeking medical assistance (25)	3,441 asthma deaths in 2018 (26) Crude death rate of 1.1 and age-adjusted death rate of 0.9 per 100,000 (13) In 2018 prevalence of asthma 24,753,379 (35) Asthma as concomitant cause of death in 10,331 deaths in 2018 (13)
Pneumonia	Delays in obtaining appropriate treatment (27)	Increased mortality and 30-day inpatient mortality (27)	Pneumonia was concomitant cause of death in 148,498 deaths in 2018 (13) 1.5 million cases of pneumonia diagnosed in ED in 2018 (28) 2018 underlying cause of death 47,956; crude death rate 14.7 and age-adjusted rate 12.0 per 100,000 (13)
Pulmonary Embolism	Delay of mean 4.2 days seeking diagnosis and care (29) Delayed mortality 30% vs. early diagnosed and treated 8% (30) Underestimating/misinterpreting chest pain/dyspnea, or attribution to other conditions (4) Mean diagnostic delay based on 12 studies was 6.3 days (38)	Mortality of 1.6% if diagnosed early, increasing to 43.2% if delayed diagnosis (31)	Annual incidence 900,000 (32) 2018 underlying cause of death in 8,809 deaths; crude death rate 2.7 and age-adjusted rate 2.3 per 100,000 (13) Concomitant cause of 36,494 deaths in 2018 (13)

In 2019, stroke accounted for 1 in 19 US deaths, the fifth leading cause of mortality (22, 33). Prehospital suspicion of stroke compressed time to computer tomography by 219 minutes and to stroke unit care by 556 minutes, reducing mortality (23, 34). However, 42.0% of patients experienced pre-hospital delays due to little knowledge of stroke symptoms (21). In the present study, 45.6% of patient-users with stroke symptoms intended to pursue non-emergency care, including 24.0% who planned to recover at home or take no action (ED overtriage adjusted). Reducing pre-hospital stroke care delay and acuity of care mismatch is a priority (21, 23). VT can potentially reduce delays in emergency stroke care.

In 2019, asthma caused 1,835,901 US ED visits, 169,330 hospital admissions, and 3,524 deaths (35–37). Many deaths and admissions were preventable and related to care delays (5, 7). In total, 45% of patients did not seek medical care during the final fatal asthma attack (25).

In 2021, 1.4 million US cases of pneumonia were diagnosed in EDs, 47,956 of which were fatal (13). Pneumonia was a concomitant cause of death in 148,498 deaths (13). Timely antibiotic treatment reduced adjusted 30-day hospital mortality by 16.0% (27).

Pulmonary embolism caused 8,809 US deaths in 2018 (13), with untreated mortality of 30.0% (30). In-hospital mortality was 1.6% for early versus 43.2% for delayed PE diagnosis (31). Treatment was delayed for 4.2 days due to patient procrastination (29), with 18–38% of patients symptomatic a week or longer before diagnosis (32). VT well integrated with healthcare system triage and intake can potentially reduce patient underestimation of symptom severity and delays in care seeking for these deadly respiratory illnesses.

Our findings that 53.5% of patients (ED overtriage adjusted) who reported symptoms of these five life-threatening conditions had no pre-triage intent to engage urgent care, and 33.5% had no intent to seek any medical care, aligns with national data. Users of VT may wish to assess their symptoms without engaging local healthcare for convenience and/or to save time and expense. Patients may seek reassurance that their symptoms are relatively benign. This sampling of 12,000 interviews from over three million total VT episodes demonstrates that in a significant percentage, possible existence of a severe underlying cause of symptoms should prompt the patient-user to seek professional medical attention sooner than they would have otherwise planned. The potential to reduce morbidity and mortality by more timely assessment of ambiguous/unrecognized signs and symptoms has significant public health implications. We suspect that use of AI-driven VT may become a valuable addition to under-resourced healthcare systems unable to assess all patients who seek care for uncertain symptoms but who may be at elevated risk for more serious disease.

With respect to limitations of the study, it is pertinent to acknowledge that the data collected may have been affected by certain inaccuracies. For instance, patient-users may have clicked through initial questions regarding their pre-VT care intention or conveyed random responses in order to get to the part of the encounter they were interested in, for example the VT evaluation of symptoms presented. While we have no evidence that this behavior occurred in substantial numbers of VT patient-users, the question warrants further research consideration and detection measures if a prevalent behavior.

In the effort to change patient care seeking behavior when confronted with potentially life threatening symptoms, interventions are needed that improve patient engagement, education, understanding of the clinical signs and symptoms of these acute conditions, and which are effective in activating patients to immediately seek emergency care. Given that many lives are avoidably lost, and the quality and extent of others are diminished, our current inability to effectively engage and educate patients to seek urgent care when confronted with life threatening symptoms is an area demanding far greater behavior change research, along with associated research funding. Indeed, one wonders what could be achieved in understanding and affecting patient care seeking behavior change were this research to be funded at the level of pharmaceutical development and marketing of a new therapeutic to treat patients whose severity of illness could have been lessened, if we were more capable of identifying and activating these patients to earlier care seeking and reduction of care delay.

Clearly, current approaches to achieving these behavioral and care seeking objectives have been largely inadequate. Public awareness campaigns addressing the importance of seeking prompt medical care in cases of acute, life-threatening cerebrovascular symptoms, for example, demonstrate a positive impact (39) but are often episodic rather than continuous, which limits their long-term impact and benefit. Reliable online medical resources exist which educate about the importance of early recognition and care seeking for serious cardiac symptoms as well, but provide general, non-personalized medical information, which can lessen the impact on individual behavior.

Subsequent research should systematically assess patient outcomes in order to fully evaluate the potential of VT to earlier detect severe evolving pathologies, such as those reported here, and effectively motivate and accelerate patient care seeking. Prospective validation in a real world clinical setting could track outcomes of patients whose self-perception and healthcare intent prior to VT is decoupled from actual acuity and urgent care need. Using objective clinical parameters and endpoints, this would assess the role and clinical value of AI-enabled VT within a care delivery and patient engagement ecosystem. If thus validated, VT may offer payers, health systems and providers a real-time vehicle for earlier detection and engagement of at-risk patients to enable better care acuity alignment and rapid ED referral/ambulance dispatch for life-threatening conditions.

## Conclusion

The impact of advanced diagnostics and therapeutics is blunted if patients do not rapidly engage appropriate acuity care to receive their benefits. This study suggests that VT AI may help accelerate the moment of disease detection and appropriate acuity level care engagement. VT lets a user know their condition may be more serious than they perceive and they should seek ED care. Integrated within a digital front door, VT can potentially accelerate early detection, care acuity alignment and live engagement/intake to save lives, reduce morbidity, and better utilize healthcare resources by reducing care delays due to patient uncertainty and/or misunderstanding. Future research should validate that VT can influence patient care seeking behavior to enable accelerated ED care referral or ambulance dispatch. If validated, VT may become an integral component in an AI-enhanced clinical information ecosystem that expedites detection and delivery of appropriate urgent medical care.

Currently, when patient care intent is decoupled from actual clinical need by patient misunderstanding or indecision, earlier care detection and accelerated care engagement to reduce care delay is a complex challenge. As remote diagnostic monitoring and telemedical capabilities advance in coming years, so too will AI-based VT. Rapid development in generative AI and large language models will deliver improved user experience of VT AI. The use of natural language to enable patient-users to communicate in a more effective and nuanced way with VT AI will enable greater sensitivity, engendering trust in the results and greater impact on patient-user behavior. Exploiting these emerging potentialities will require investment and multisector scientific expertise converging private industry and public-governmental interests, assets, and capabilities.

The VT industry is not sufficiently resourced to drive the research needed to explore the potential beneficial impact and value of AI-based VT in early detection and acuity-aligned care referral. Furthermore, population databases of what will soon be hundreds of millions of VT episodes across the industry are too valuable a data and research resource to be applied exclusively for purely commercial interest. The VT industry should enable access to its anonymized aggregate data by the US National Institutes of Health and Centers for Disease Control and Prevention, international agencies, academic medical centers, and healthcare organizations to investigate and progress the full potential value of VT in the emerging 21st century health IT and AI ecosystem.

## Data availability statement

The raw data supporting the conclusions of this article will be made available by the authors upon reasonable request.

## Ethics statement

The studies were conducted in accordance with the local legislation and institutional requirements. The participants provided their written informed consent to participate in this study.

## Author contributions

GAG: Conceptualization, Data curation, Formal analysis, Investigation, Methodology, Project administration, Validation, Visualization, Writing – original draft, Software, Supervision, Writing – review & editing. AK-K: Conceptualization, Data curation, Formal analysis, Methodology, Validation, Writing – review & editing. GLG: Data curation, Project administration, Writing – review & editing. JR-S: Investigation, Writing – review & editing. SG: Conceptualization, Formal analysis, Resources, Writing – original draft, Writing – review & editing. TP: Investigation, Project administration, Supervision, Writing – review & editing. KK: Data curation, Project administration, Writing – review & editing. NM: Writing – original draft. MP: Conceptualization, Writing – original draft. Writing – review & editing. JJ: Conceptualization, Writing – original draft, Writing – review & editing. IL: Writing – review & editing. PO: Project administration, Supervision, Writing – review & editing.
